# Breaking symmetry: effects of habitat disturbance on flight-related traits of two Triatominae species

**DOI:** 10.3389/finsc.2025.1651021

**Published:** 2025-09-08

**Authors:** Federico G. Fiad, Julieta Nattero, Miriam Cardozo, Gisel V. Gigena, Ana López, Fernando Carezzano, David E. Gorla, Claudia S. Rodríguez

**Affiliations:** ^1^ Cátedra Morfología Animal, Facultad de Ciencias Exactas, Físicas y Naturales Universidad Nacional de Córdoba, Córdoba, Argentina; ^2^ Departamento de Ecología Genética y Evolución, Laboratorio de Eco-Epidemiología, Facultad de Ciencias Exactas y Naturales, Universidad de Buenos Aires, Ciudad Autónoma de Buenos Aires, Buenos Aires, Argentina; ^3^ Instituto de Ecología, Genética y Evolución (IEGEBA), CONICET-Universidad de Buenos Aires, Ciudad Autónoma de Buenos Aires, Buenos Aires, Argentina; ^4^ Departamento de Biodiversidad y Biología Experimental. Facultad de Ciencias Exactas y Naturales. Universidad de Buenos Aires, Ciudad Autónoma de Buenos Aires, Buenos Aires, Argentina; ^5^ Cátedra Introducción a la Biología, Facultad de Ciencias Exactas, Físicas y Naturales Universidad Nacional de Córdoba, Buenos Aires, Argentina; ^6^ Instituto de Investigaciones Biológicas y Tecnológicas (IIBYT-CONICET), Córdoba, Argentina; ^7^ Instituto de Diversidad y Ecología Animal (IDEA-CONICET), Córdoba, Argentina

**Keywords:** adaptation, dispersion, fluctuating asymmetry, habitat fragmentation, *Triatoma garciabesi*, *Triatoma guasayana*

## Abstract

**Introductiom:**

Habitat fragmentation alters environmental structure and imposes selective pressures on dispersal-related traits in insect vectors, potentially driving morphological adaptations that enhance flight performance. In this study, weinvestigate how landscape metrics influence the size and shape of the head and wings in two Triatominae species, *Triatoma garciabesi* and *T. guasayana*, which present differing ecological strategies. We hypothesize that individuals from more fragmented landscapes exhibit phenotypic shifts associated with enhanced dispersal capacity and increased morphological symmetry.

**Methods:**

To test this, we combined community-based sampling of triatomines with geometric morphometrics and multiscale landscape metrics. We applied geometric morphometrics and generalized linear models (GLM)-based analyses to assess the effects of habitat fragmentation on flight-related morphology.

**Results:**

Our results reveal that *T. garciabesi* shows increased head asymmetry and narrower wings in highly fragmented landscapes, while *T. guasayana* exhibits subtle shifts in head shape asymmetry and greater sexual dimorphism. In both species, head and wing sizes tended to be larger in fragmented habitats, especially in females, suggesting differential morphological responses that may reflect species-specific dispersal strategies.

**Discussion:**

Habitat fragmentation differentially affects *T. garciabesi* and *T. guasayana*, leading to distinct dispersal syndromes. *Triatoma garciabesi* shows greater plasticity, highlighting the role of landscape structure in shaping adaptive dispersal traits.

## Background

Habitat fragmentation caused by human activities in natural environments, altering ecosystem characteristics such as area reduction, loss of vegetation, changes in microclimatic conditions and the isolation of remaining vegetation fragments (1, 2). These changes reduce the size of habitat patches, supporting smaller populations and increasing the risk of stochastic extinction (3). Furthermore, greater inter-patch distances increase dispersal challenges, potentially intensifying the extinction vortex (3, 4). These effects are further exacerbated by deforestation and global temperature increases, significantly modifying the ecology and behaviour of many organisms (5). Some studies on insects involved in disease transmission show increased prevalence in deforested areas (6–8). However, other studies suggest that forest conservation could boost vector abundance, potentially enhancing their invasion of human environments (9, 10). These contrasting conclusions may arise from differences in the ecological dynamics, life history, and phenotypic traits of insect vectors, which exhibit distinct dispersal behaviours, habitats, and interactions with their environments.

Dispersal refers to the movement from birth to breeding sites and involves three stages: emigration, transience, and immigration (11–14). Its evolution balances the costs and benefits at each stage, which are shaped by individual, social, and environmental factors (13, 15–17). Morphological phenotypic traits underlying dispersal are classified as enabling traits (necessary for dispersal), enhancing traits (which reduce costs or improve efficiency), and matching traits (which facilitate non-random movements) (3). In fragmented landscapes, dispersal is further challenged by increasing distances between habitat patches, requiring traits that improve efficiency and reduce costs (18). Adverse developmental environments can amplify genetic variation or reduce developmental stability, increasing phenotypic plasticity and enabling individuals to adapt to fragmented habitats. This plasticity may lead to genetic changes through genetic assimilation or accommodation (19–22).

Head and wing size and shape are key determinants of dispersal in insects and influence flight performance, orientation, and energy efficiency (18, 23–25). Morphological variation in these traits can mediate responses to selective pressures imposed by habitat fragmentation, promoting the evolution of forms that optimize flight in open or structurally complex environments. For instance, narrower wings may reduce energetic costs during long-distance flight, while changes in head morphology may relate to enhanced sensory processing or aerodynamics (23, 24, 26). These associations between form and function suggest that morphological traits may reflect adaptations to environmental structure and provide insight into dispersal dynamics in changing landscapes (3).

Symmetry in biological structures, a fundamental feature controlled by the genome, reflects developmental stability. In contrast, deviations from symmetry, such as fluctuating asymmetry (FA), indicate an organism’s capacity to buffer environmental variability and maintain consistent phenotypic expression (27, 28). Together, these mechanisms highlight how phenotypic traits and their plasticity mediate dispersal processes in fragmented habitats, providing insights into the evolutionary dynamics of species. Specifically, developmental stability buffers environmental variation and ensures consistent phenotypic expression within individuals possessing a particular genotype and environment (29). Thus, research on developmental stability along an anthropization gradient represents a valuable approach to understanding how environmental modification influences the dispersive characteristics of an insect species. To our knowledge, this is the first study using this approach in Triatominae species (review in 30).

This study focuses on *Triatoma garciabesi* Carcavallo, Cichero, Martinez, Prosen & Ronderos, 1967 and *T. guasayana* Wygodzinsky & Abalos, 1949 (Hemiptera: Reduviidae, Triatominae), secondary vectors of *Trypanosoma cruzi* Chagas, 1909 (Kinetoplastida, Trypanosomatidae), the etiological agent of Chagas disease. These species sustain the parasite transmission cycles in sylvatic environments and may connect wild and domestic cycles (31, 32). Both are spread across Argentina, Bolivia, and Paraguay (33, 34). *Triatoma garciabesi*, an arboreal species linked to birds, inhabits the loose bark of *Prosopis* sp. and maintains high population densities year-round (35). In contrast, *T. guasayana*, a terrestrial species associated with mammals, resides in dry cacti, fallen logs, and bromeliads, with population densities declining in winter (36, 37). Both species are known to invade rural houses during summer in the western Chaco Seco ecoregion of Argentina (35, 38–42).

Habitat degradation caused by human activities affects triatomine populations, promoting their dispersal into artificial ecotopes (43). Host populations decline and habitat loss impose selective pressures on flight-related traits, potentially enhancing mobility in dynamic landscapes (3, 44). For these two species, a recent study demonstrates that flight-dispersal characteristics changed in response to varying degrees of anthropization (45). These findings may be linked to the direct impact of fragmentation on the phenotypic specialization hypothesis, which proposes that landscape fragmentation exerts selective pressure on populations. Combined with greater inter-patch distances, this pressure may drive the evolution of alternative dispersal strategies (3).

In this research, we aim to understand how landscape fragmentation acts as a selective pressure on the phenotypic plasticity of flight-related traits in two species of triatomines. Particularly, our goal was to determine the effects of landscape metrics on changes in the shape and size of the head and wings in both species of triatomines. We hypothesize that habitat fragmentation promotes phenotypic shifts in flight-related traits in *T. garciabesi* and *T. guasayana*, favouring morphologies associated with enhanced dispersal capacity. Moreover, these phenotypic changes are shaped according to the life-history characteristics of each species, which could lead to different responses depending on their reproductive strategies, life cycles, and ecological adaptations. Specifically, we expect individuals from more fragmented landscapes to exhibit larger head and wings and particular shape modifications that improve aerodynamic efficiency. Moreover, we predict that morphological symmetry will be higher in these populations, suggesting directional selection towards optimal flight performance rather than increased developmental stress.

## Methods

### Study area and insect collection

**Figure 1:**
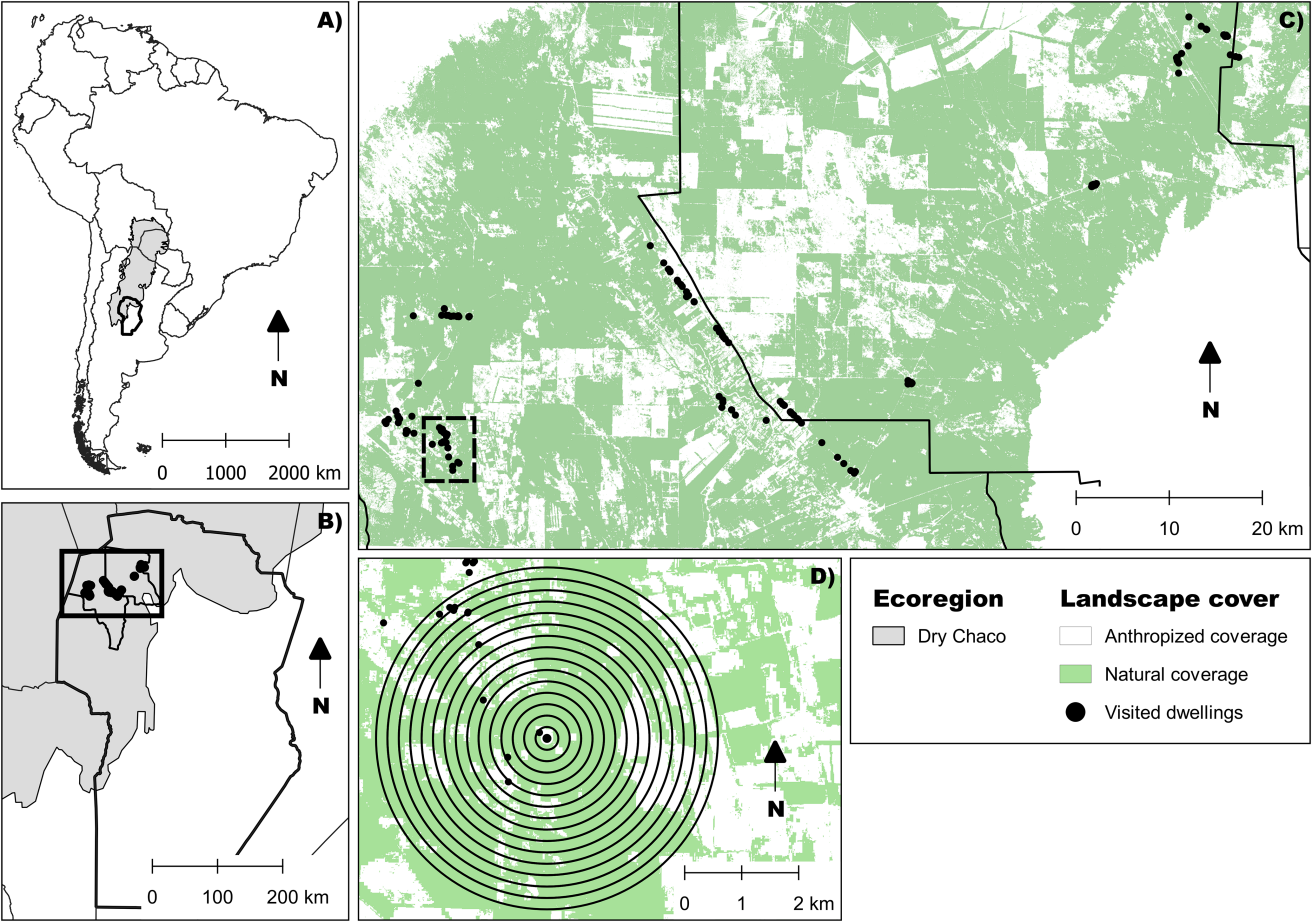
**(A)** Location of the Dry Chaco Ecoregion in South America (shaded in grey). **(B)** Study area located in the departments of Cruz del Eje and Ischilín, in the northwest of Córdoba Province, Argentina. **(C)** Characterization of anthropogenic environmental disturbance based on supervised land-cover classification (45). Black points indicate rural dwellings where triatomines were collected. **(D)** Landscape characterization around each dwelling. Concentric circles represent the spatial buffers used for landscape metric extraction, with diameters ranging from 200 to 3000 meters.

The study was conducted between 2017 and 2020, during the warm season’s beginning (October to December) and ending (February to March). Sampling occurred in 131 dwellings across 14 rural communities in the Cruz del Eje and Ischilín departments (northwest Córdoba, central Argentina), situated at the southern edge of the Gran Chaco region (**Figure 1**). All dwellings were georeferenced using a GPS device (Garmin Etrex 30) and assigned a unique code. This selected region, part of the Arid Chaco, has historically been endemic for Chagas disease and is characterized by a semi-desert climate with annual precipitation ranging from 400 to 700 mm, mostly concentrated in summer. The area experiences hot summers with temperatures exceeding 40°C and cold winters with lows below 5°C (46–48). The terrain consists of gently rolling plains with saline-alkaline soils and diverse natural vegetation, including *Aspidosperma quebracho-blanco* Schltdl., 1861, *Prosopis* sp. L., 1767, *Celtis* sp. L., 1753, and *Geoffroea decorticans* (Gillies ex Hook. & Arn.) Burkart, 1949 forests and shrublands dominated by species such as *Mimozyganthus carinatus (*Griseb.) Burkart, 1939 and *Larrea divaricate* Cav., 1800 (49). Land use in the region includes extensive livestock farming and the cultivation of various crops, including vegetables, fruits, and grains (50).

Previous research defined an anthropization gradient with three levels (45). This gradient was established by classifying land cover into two categories: natural (including forests, shrublands, and water bodies) and artificial (comprising bare soil, managed pastures, and crops). The expansion of the agricultural frontier in this region has resulted in varying levels of anthropization, ranging from highly modified agricultural landscapes to areas with minimally disturbed natural environments. Highly anthropized areas are characterized by replacing natural vegetation with crops and pastures, while intermediate areas exhibit a mosaic of human activity and natural vegetation fragments. Low-anthropization areas largely preserve native ecosystems. In some regions, poor soil conditions and water scarcity have constrained agricultural expansion, leading to lower levels of human intervention.

Community-based vector surveillance was employed to capture triatomines in the study area (42). During fieldwork, each household owner received a plastic bag to collect triatomines found inside their homes. After 15 days, the bags were retrieved to gather the specimens. In the laboratory, the collected triatomines were taxonomically identified, sexed, photographed, dissected, and preserved in 70% alcohol. Taxonomic identification followed the keys of Jurberg et al. (51) and Lent and Wygodzinsky (52). For this study, only 155 *T. garciabesi* and 331 *T. guasayana* collected at the beginning of the summer season were considered to minimize seasonal variation (53).

### Landscape fragmentation and the influence on phenotypic variation

Metrics were analyzed to evaluate the relationship between landscape features and phenotypic traits. Flight-dispersal traits in *T. garciabesi* and *T. guasayana* are known to be influenced by anthropization pressures (45). Landscape fragmentation can increase dispersal time and energy costs, reduce success, and highlight the importance of patch geometry and matrix suitability (3, 45, 54). Percentage of natural vegetation cover (PLAND), number of patches (NP), landscape shape index (LSI), and aggregation index (AI) were selected to assess their influence on phenotypic changes. FRAGSTATS v4.2.598 was used to calculate these metrics based on a natural-anthropic land cover map derived from supervised classification (45). Landscape metrics were extracted at 15 scales, represented by buffers with diameters ranging from 200 to 3000 meters around each dwelling, using QGIS 3.26.2 (**Figure 1**).

### Geometric morphometrics and asymmetry assessment

**Figure 2:**
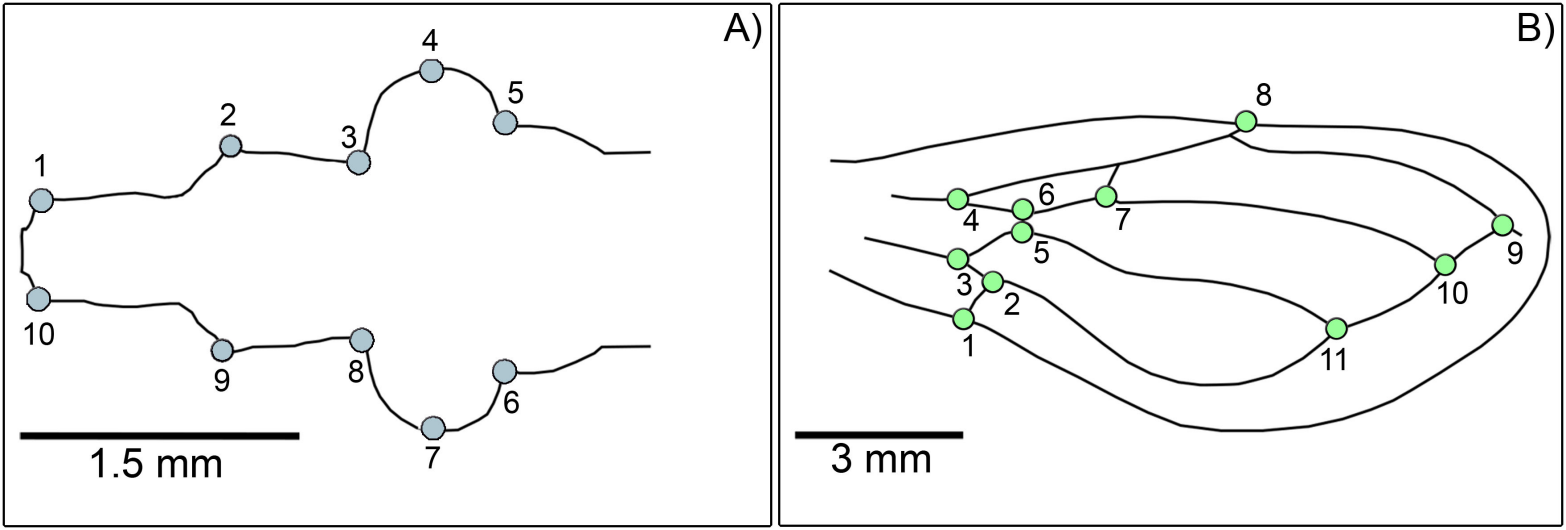
Representation of type II morphological landmarks and type I landmarks identified on the head and wings of *Triatoma garciabesi* and *T. guasayana*. **(A)** Head: 1 and 10, right and left extremities of the clypeus; 2 and 9, the base of the antenniferous tubercle; 3 and 8, the anterior base of the compound eye; 4 and 7, maximum curvature of the compound eyes; 5 and 6, the posterior base of the compound eyes. **(B)** Wings: 1, intersection of postcubitus (Pcu) and Pcu + first anal vein; 2, intersection of Pcu and cubitus (Cu) - Pcu; 3, intersection of Cu and Cu - Pcu; 4, bifurcation of radial (R) and media (M) veins; 5, intersection of Cu and M - Cu; 6, intersection of M - Cu; 7, on vein M; 8, on the subcostal vein (Sc) at the wing’s extreme edge; 9, intersection of veins R and Cu; 10, intersection of vein M and the extension of veins Cu - Pcu; 11, intersection of Pcu and Cu.

We employed landmark-based geometric morphometrics to quantify variations in head and wing shapes (55). High-resolution photographs of the dorsal view of the head and both sides of the wings of all individuals were captured using a Zeiss SV 11 stereomicroscope paired with an Olympus VG 160 digital camera. Ten landmarks on the head and eleven on each side of the wings were digitized using tpsDIG v2.32 (56) (**Figure 2**). A landmark recording was performed twice for each individual to evaluate digitizing errors. Specimens with damaged structures that impeded accurate landmark placement were excluded from the analysis.

Landmark coordinates were reflected to generate mirror images, obtaining shape and size variables for the head and wings (57). Generalized Procrustes analysis was then used to superimpose all configurations (58). For the head, this process allowed the separation of symmetric and asymmetric variation components through object symmetry analysis. For the wings, the right and left configurations were compared, treating each side as a separate configuration (57).

### Statistical analysis

To test for directional asymmetry (DA) and FA while accounting for measurement error, Procrustes ANOVA was performed (57). The variation among individuals, corrected for asymmetry effects, represented the symmetric component. The variation due to differences between the right and left sides indicated DA, while the individual × reflection interaction captured FA, reflecting variability in right-left differences among individuals. Procrustes superimposition and ANOVA were carried out using the *gpagen* and *bilat.symmetry* functions, respectively, from the *geomorph* package in RStudio (59).

Then, Procrustes ANOVA and pairwise comparisons were performed to assess differences in the shape components of the head and wings with anthropization levels. These results allowed us to identify the size and shape variables related to landscape metrics in the species’ heads and wings. The size and shape variables that showed significant differences across anthropization levels were used to develop the models.

From the selection of response variables, Partial Least Squares (PLS) analyses were conducted to identify the scale at which fragmentation most effectively explained phenotypic variation. This was determined by the strength of the relationship between landscape metrics and phenotypic traits, including symmetric and asymmetric shape components and centroid size variations in both triatomine species. Before the analysis, each landscape metric was standardized to have a mean of zero and a standard deviation of one. Variables that did not show significant effects in the Procrustes ANOVA or the comparisons across anthropization levels were excluded from further analyses. This decision was made because no statistical evidence was found to support a relationship between morphological changes in shape or size and the landscape metrics analyzed.

To test the relationship between landscape fragmentation and changes in shape and size components of flight dispersal traits in both species of triatomines, we performed two different approaches due to the differences in dimensions of the response variables. To avoid multicollinearity between variables, the variance inflation factors (VIFs) were calculated for each model, discarding the models with a VIF value > 5 (60). A Procrustes regression analysis was performed using the function *procD.lm* from the *geomorph* R package for the multidimensional shape components. This function allowed us to assess statistical hypotheses describing patterns of shape variations for a set of Procrustes shape variables. To evaluate statistical significance, a permutation-based Procrustes ANOVA using residual randomization was employed; this analysis provides a robust evaluation of effects by generating pseudo-values through the permutation of residuals relative to the fitted model, preserving the data structure (61). Additionally, a multi-model inference approach based on the Akaike information criterion (AIC) was applied to estimate the effects of predictors and their relative importance on the response variables (60). AIC scores were calculated using log-likelihoods, incorporating parameter penalties. The parameter penalty was based on the model’s number of parameters and the covariance matrix’s dimensions. This comprehensive approach allowed for a robust evaluation of model performance while accounting for the multivariate structure of the data.

For the unidimensional response variable, the centroid size (CS) of the study structures. The relationship between CS and the landscape metrics was tested using a generalized linear model (GLM). A lognormal distribution function and an identity link were used. The selection of models was based on AIC by comparing nested models following a backwards procedure (60). The assumptions of normality, homogeneity and independence in the residuals were met in all the models. The significant p-value was set at *p* < 0.05.

## Results

### Overall patterns of symmetry and asymmetry concerning anthropization levels in *Triatoma garciabesi* and *T. guasayana*

As shown in **Table 1**, the Procrustes ANOVA results revealed distinct patterns of symmetry and asymmetry concerning symmetry (individual factor), DA, and FA in *T. garciabesi* and *T. guasayana*. In *T. garciabesi*, the results indicated that the head shape was statistically significant in FA variation. Symmetry and FA were the main sources of wing shape and size variation. While in *T. guasayana*, head shape, wing shape, and size variation were statistically significant in symmetry, DA, and FA.

**Table 1:** Results of Procrustes ANOVA for the effects of individual, side, and their interaction on the shape of the head and wing and wing size in *Triatoma garciabesi* and *T. guasayana*.

Species	Module		Factor	Df	SS	F	*p*-value
*Triatoma garciabesi*	Head	Shape	Individual	94	0.135	1.88	0.079
			Side	1	0.001	1.661	0.002^**^
			Individual x side	94	0.071	6.887	0.0001^***^
			Error	190	0.021		
	Wing	Shape	Individual	93	0.158	4.443	0.0010^**^
			Side	1	0.0002	0.602	0.083
			Individual x side	93	0.035	5.497	0.0001^***^
			Error	188	0.013		
		Size	Individual	93	1.05e-05	10.704	0.0001 ^***^
			Side	1	6.90e-09	0.652	0.228
			Individual x side	93	3.65e-06	3.725	0.0001 ^***^
			Error	188	1.98e-06		
*Triatoma guasayana*	Head	Shape	Individual	261	0.28	1.868	0.007^**^
			Side	1	0.0007	1.223	0.0003^**^
			Individual x side	261	0.15	6.97	0.0001^***^
			Error	524	0.043		
	Wing	Shape	Individual	287	0.476	4.308	0.0001^***^
			Side	1	0.0007	1.796	0.004^*^
			Individual x side	287	0.11	6.891	0.0001^***^
			Error	576	0.032		
		Size	Individual	287	2.98e-05	20.282	0.0001 ***
			Side	1	1.76e-07	34.33	0.0001 ***
			Individual x side	287	1.45e-05	9.851	0.0001 ***
			Error	576	2.95e-06		

*Df*, degrees of freedom; *SS*, sum of squares; *F*, Goodall F ratio; *Z*, Z-score. *p<0.05, **p<0.005, ***p<0.001.

**Table 2** presents the summary statistics for the pairwise comparison of symmetry and asymmetry in head and wing shape across levels of anthropization for *T. garciabesi* and *T. guasayana*. The results of head shape asymmetry for the first species revealed differences between intermediate and low levels of anthropization, with the low level of anthropization exhibiting lower FA values. Furthermore, the symmetric component of wing shape differed significantly between intermediate and low levels of anthropization. Regarding the second species, head shape symmetry displayed significant differences between high and low anthropization levels and intermediate and low levels. Head and wing shape asymmetry also showed significant differences between low and intermediate levels of anthropization, however, the observed FA differences between anthropization levels were subtle. Based on these results, only those terms showing significant effects were analyzed further to assess whether they varied across levels of anthropization (**Table 2**).

**Table 2:** Pairwise comparisons of shape symmetry and asymmetry in the head and wing of *Triatoma garciabesi* and *T. guasayana* across levels of anthropization.

Species	Module	Shape component	Level of anthropization	Distance between vectors	Upper Confidence Limit (UCL 95%)	*p*-value
*Triatoma garciabesi*	Head	Symmetry	HI	0.77	1.09	0.378
			HL	0.57	0.99	0.678
			IL	0.42	0.77	0.731
		Asymmetry	HI	0.73	1.31	0.719
			HL	0.87	1.19	0.357
			IL	0.97	0.94	0.037*
	Wing	Symmetry	HI	1.17	1.25	0.083
			HL	0.98	1.15	0.174
			IL	1.06	0.89	0.006**
		Asymmetry	HI	0.56	0.89	0.627
			HL	0.66	0.83	0.252
			IL	0.38	0.64	0.703
*Triatoma guasayana*	Head	Symmetry	HI	0.42	0.49	0.163
			HL	0.53	0.51	0.036*
			IL	0.71	0.40	0.0001***
		Asymmetry	HI	0.54	0.60	0.112
			HL	0.61	0.62	0.057
			IL	0.60	0.48	0.004**
	Wing	Symmetry	HI	0.27	0.65	0.967
			HL	0.48	0.66	0.364
			IL	0.44	0.49	0.120
		Asymmetry	HI	0.36	0.46	0.286
			HL	0.28	0.47	0.728
			IL	0.40	0.35	0.015*

Comparison groups between levels of anthropization.

HI, High level vs Intermediate level; HL, High level vs Low level; IL, Intermediate level vs Low level.

*p<0.05, **p<0.005, ***p<0.001.

### Effective spatial scales for morphological responses to landscape fragmentation

Overall, the results obtained from the PLS analyses indicated different patterns between the species and the shape and size components. The asymmetric shape (**Supplementary Figures S1A-C**) and size (**Supplementary Figures S1D-F**) of the head, as well as the symmetric shape (**Supplementary Figures S1G-I**) and the size of the wing (**Figurea S1J-L**) of *T. garciabesi* were associated with landscape metrics measured at distances ranging from 600 to 3000 meters from the dwellings. Moreover, the comparison between the effect size of the PLS from 600 to 3000 meters showed no significant differences, indicating that the shape and size components were not scale-dependent. The distances at 200 and 400 meters showed no significant association, meaning that the shape and size components of the head and wings indicate little relationship with landscape predictors at those scales. Compared to *T. garciabesi*, the asymmetric head shape component of *T. guasayana* begins to be associated with landscape metrics from 1400 to 3000 meters (**Supplementary Figures S2A-C**), while the symmetric component shows an association from 2400 to 3000 meters (**Supplementary Figures S2D-F**). The comparison between the effect size of the PLS in both asymmetrical and symmetrical components of head shape showed no significant differences. There was no evidence that the landscape metrics had an influence on the centroid size (CS) of the head (**Supplementary Figures S2G-I**), asymmetry (**Supplementary Figures S2J-L**), and CS of the wing (**Supplementary Figures S2M-O**). Given the flight-dispersal capacity ranging from 200 to 2000 meters of the triatomines (62, 63) and the results presented in this section, we defined the effect size at 2000 meters to *T. garciabesi* and 2400 meters to *T. guasayana*.

### Symmetric and asymmetric morphological responses to landscape metrics in *Triatoma garciabesi*

**Figure 3:**
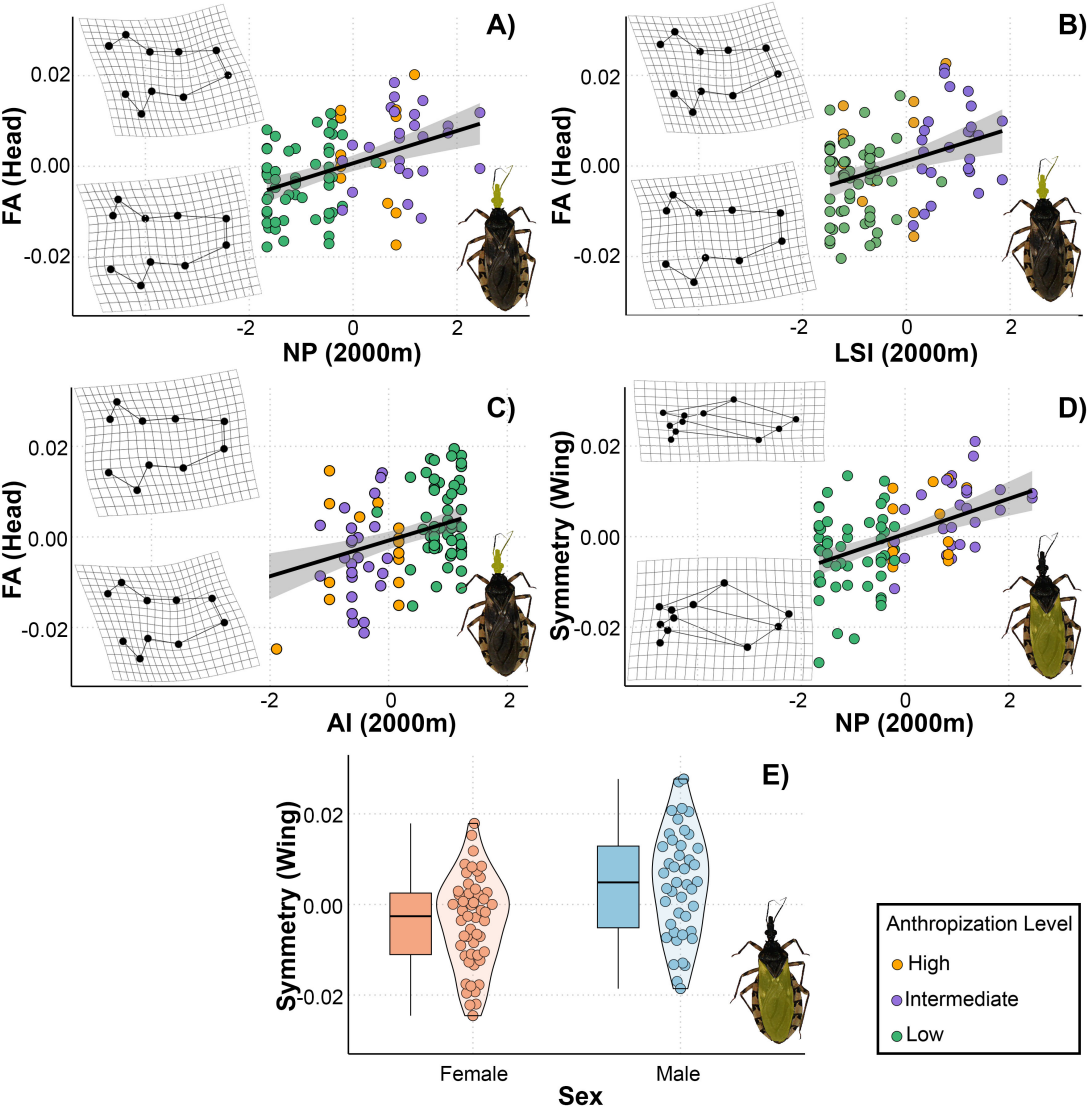
Relationship between landscape metrics at 2000 meters through an anthropization gradient on the symmetric and asymmetric shape of the head **(A–C)** and wings **(D, E)** of *Triatoma garciabesi*. Deformation grids illustrate the shape changes in the corresponding structures (head or wings) along the gradient of each explanatory variable. NP, number of patches; LSI, landscape shape index; AI, aggregation index; sex, individual’s sex.

From the total set of models for *T. garciabesi*, we selected 16 for head shape and size and wing shape and size, as these models had a VIF < 5. Three candidate models for the asymmetric head shape component and two for the symmetric wing shape component had ΔAIC < 3 (**Table 3**). The top-ranked models for asymmetric head shape included the effects of landscape configuration (AI, LSI) and composition (NP) measured at 2000 meters. Overall, head shape asymmetry increased with higher number of patches and landscape shape index but decreased with aggregation index (**Figures 3A-C**). The highest level of asymmetry was observed in the compound eyes, with greater deformation occurring in landscapes with numerous patches, higher landscape shape index, and lower aggregation index. Meanwhile, the best-ranked models for symmetric wing shape included sex and number of patches as predictor variables (**Figures 3D, E**). Wings were broader and more rounded in landscapes with fewer patches, whereas in landscapes with numerous patches, they were narrower and more pointed.

**Table 3:** Results of model selection (ΔAIC) for the effects of landscape metrics and sex on the symmetric and asymmetric head and wing shape components in *Triatoma garciabesi* and *T. guasayana*.

Species	Module	Response variable	Model	ΔAIC	R2	*p*-value	Model predictors
*Triatoma garciabesi*	Head	Asymmetry	M4	0	0.21	0.009**	AI2000m
			M3	1.32	0.18	0.023*	LSI2000m
			M2	2.24	0.23	0.016**	NP2000m
		CS	M14	0	0.60	0.0001***	LSI2000m + Sex
			M15	0.03	0.60	0.0001***	AI2000m + Sex
			M12	0.09	0.60	0.0001***	NP2000m + Sex
	Wing	Symmetry	M5	0	0.16	0.035*	Sex
			M2	0.03	0.26	0.04*	NP2000m
		CS	M12	0	0.54	0.0001***	NP2000m + Sex
			M18	2.00	0.53	0.0001***	NP2000m + PLAND2000m + Sex
*Triatoma guasayana*	Head	Symmetry	M2	0	0.09	0.0001***	NP2400m
			M5	1.09	0.28	0.0001***	Sex
		Asymmetry	M1	0	0.09	0.04*	PLAND 2400m
			M2	0.01	0.09	0.04*	NP2400m
			M4	0.03	0.10	0.028*	AI2400m
			M3	0.04	0.10	0.027*	LSI2400m
		CS	M5	0.01	0.40	0.0001***	Sex
	Wing	Symmetry	M5	0	0.17	0.0001***	Sex
		CS	M5	0	0.46	0.0001***	Sex

NP, number of patches; LSI, landscape shape index; AI, aggregation index; PLAND, percentage of landscape covered by patches; sex, individual’s sex.

*p<0.05, **p<0.005, ***p<0.001.

**Figure 4:**
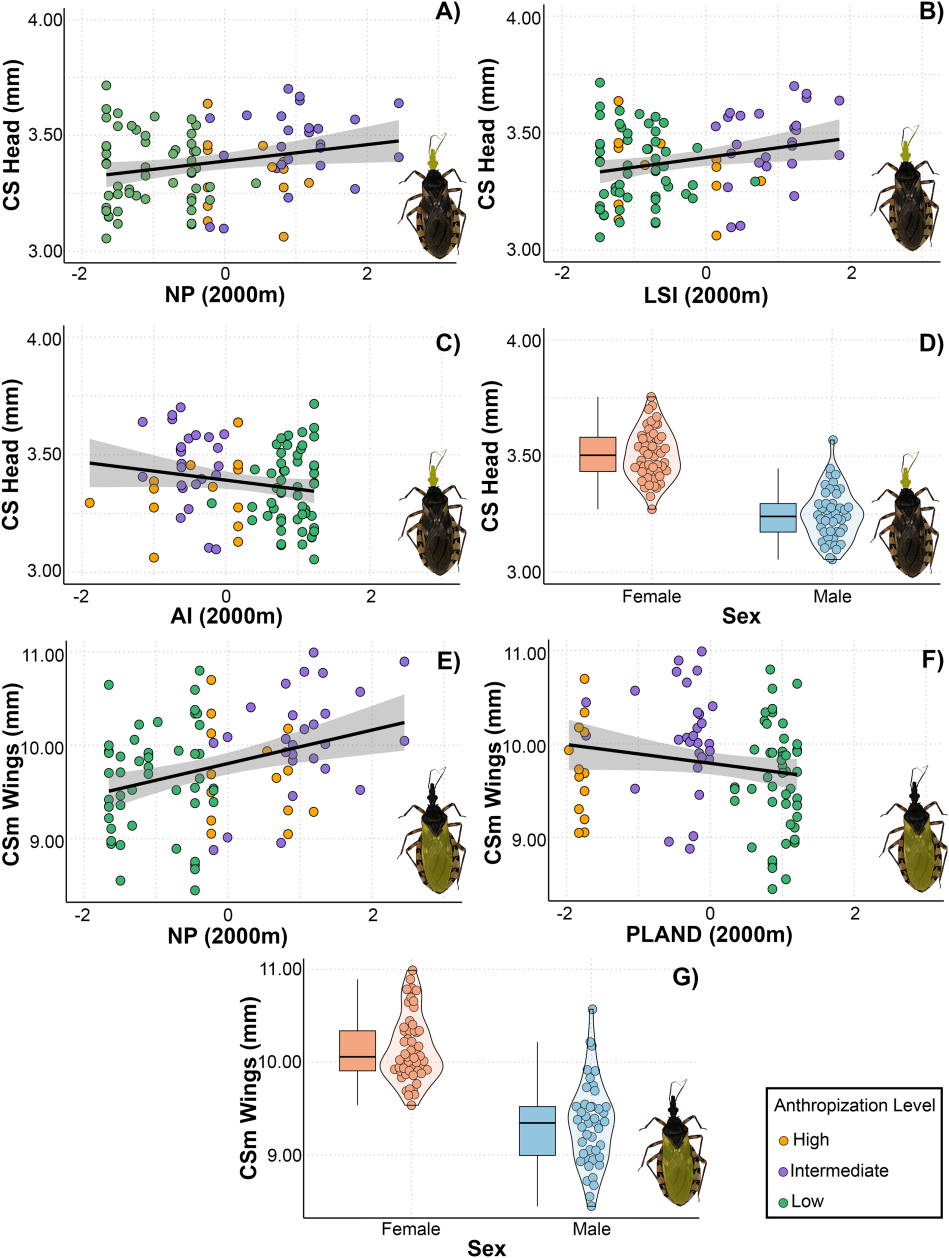
Relationship between landscape metrics at 2000 meters through an anthropization gradient on the centroid size (CS) of the head **(A–D)** and wings **(E–G)** of *Triatoma garciabesi*. NP, number of patches; LSI, landscape shape index; AI, aggregation index; PLAND, percentage of landscape covered by patches; sex, individual’s sex.

As with the asymmetric component of the head, three models with ΔAIC < 3 were selected for the centroid size of the head (**Table 3**). The top-ranked models included numer of patches, aggregation index, landscape shape index, and sex as factors associated with changes in CS. We observed a positive linear relationship between CS and number of patches and landscape shape index, suggesting that head size tends to be larger in landscapes with numerous patches of complex shapes (**Figure 4**). Additionally, there was a negative relationship between head CS and aggregation index. Regarding the CS of the wing, the top-ranked models included number of patches, PLAND, and sex as predictor variables. The model revealed a positive linear relationship between number of patches and CS, indicating that wing size tends to be larger in landscapes with numerous patches. Additionally, female wings were larger than those of males. However, the model did not reveal a significant relationship between wing size and PLAND (**Figure 4**).

### Symmetric and asymmetric morphological responses to landscape metrics in *Triatoma guasayana*

**Figure 5:**
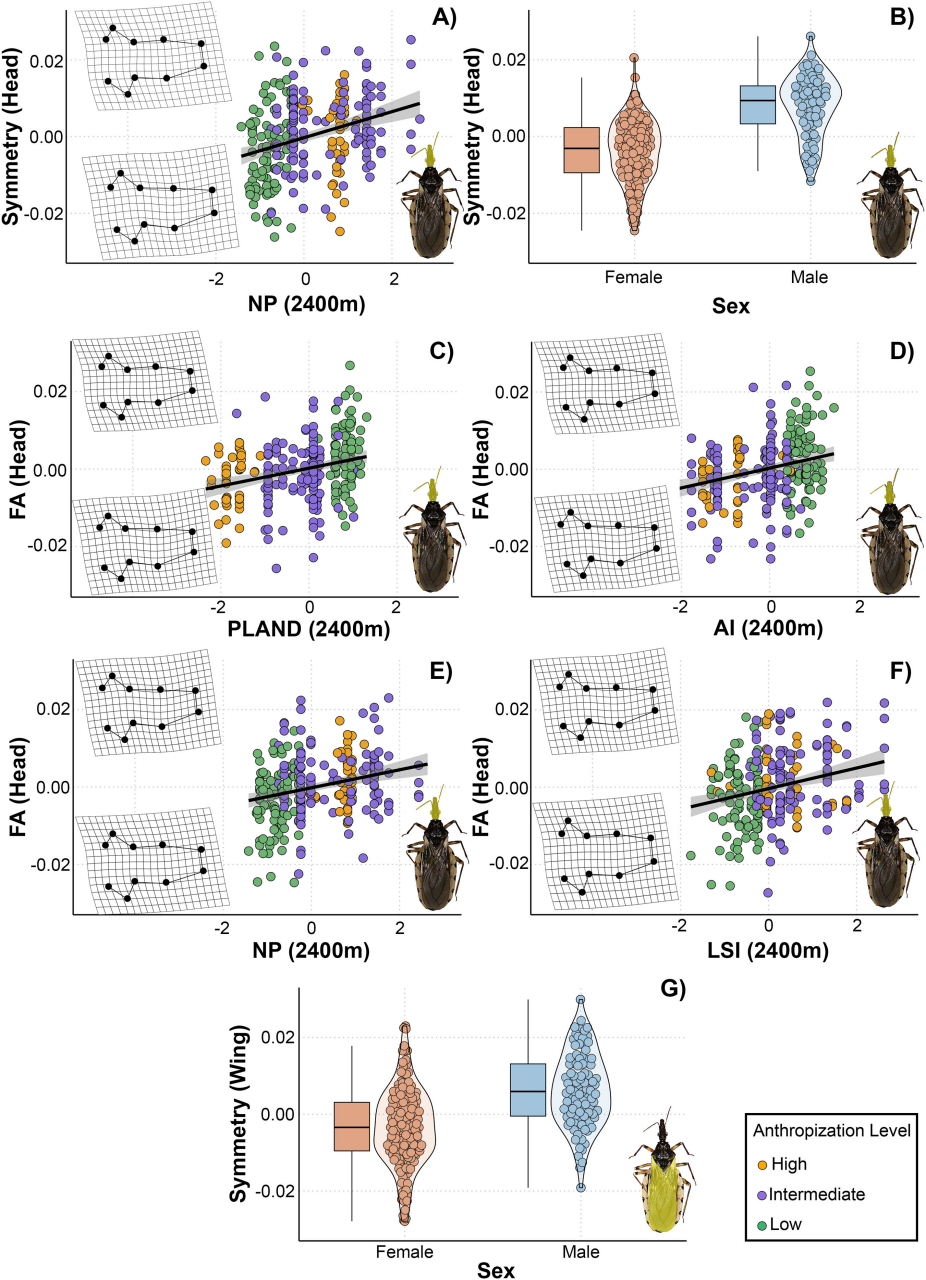
Relationship between landscape metrics at 2400 meters through an anthropization gradient on the symmetric and asymmetric shape of the head **(A–F)** and wings **(G)** of *Triatoma guasayana*, obtained from the set of best-supported models under a multimodel inference approach. Deformation grids illustrate the shape changes in the corresponding structures (head or wings) along the gradient of each explanatory variable. NP, number of patches; LSI, landscape shape index; AI, aggregation index; PLAND, percentage of landscape covered by patches; sex, individual’s sex.

The VIF calculated for the models allowed us to select 24 models for head shape and size and 21 for wing shape and size. Six models with ΔAIC < 3 were selected for the head shape: two for the symmetric component and four for the asymmetric component (**Table 3**). Only one model was selected for a symmetric wing shape. The top-ranked model for the symmetric head shape included number of patches and sex, while the models of asymmetric head shape contained PLAND, number of patches, aggregation index and landscape shape index as predicted variables. All the landscape metrics were measured at 2400 meters in this case. For symmetric head shape, the models indicated a positive linear relationship with number of patches (**Figure 5A**). In landscapes with few patches, the head shape was elongated in the anteroposterior direction and wider laterally. In contrast, in landscapes with numerous patches, it became more compressed in both the anteroposterior and lateral directions. Furthermore, we observed differences between sexes in the symmetric head shape (**Figure 5B**).

The models revealed a positive linear relationship for asymmetric head shape with PLAND, number of patches, aggregation index, and landscape shape index measured at 2400 meters (**Figures 5C-F**). The shapes derived from the models exhibited subtle changes in asymmetry, primarily in the region extending from the clypeus to the base of the compound eyes. Head asymmetry was low in landscapes with higher PLAND and aggregation index values and lower number of patches and landscape shape index values. Conversely, head asymmetry was greater in landscapes with lower PLAND and aggregation index values and higher number of patches and landscape shape index values. In the highest recorded asymmetry values, slight deformations were observed in the region mentioned above, shifting towards the right and generating a head with a slight curvature. Meanwhile, the best-ranked model for symmetric wing shape included only sex as a predictor variable (**Figure 5G**).

**Figure 6:**
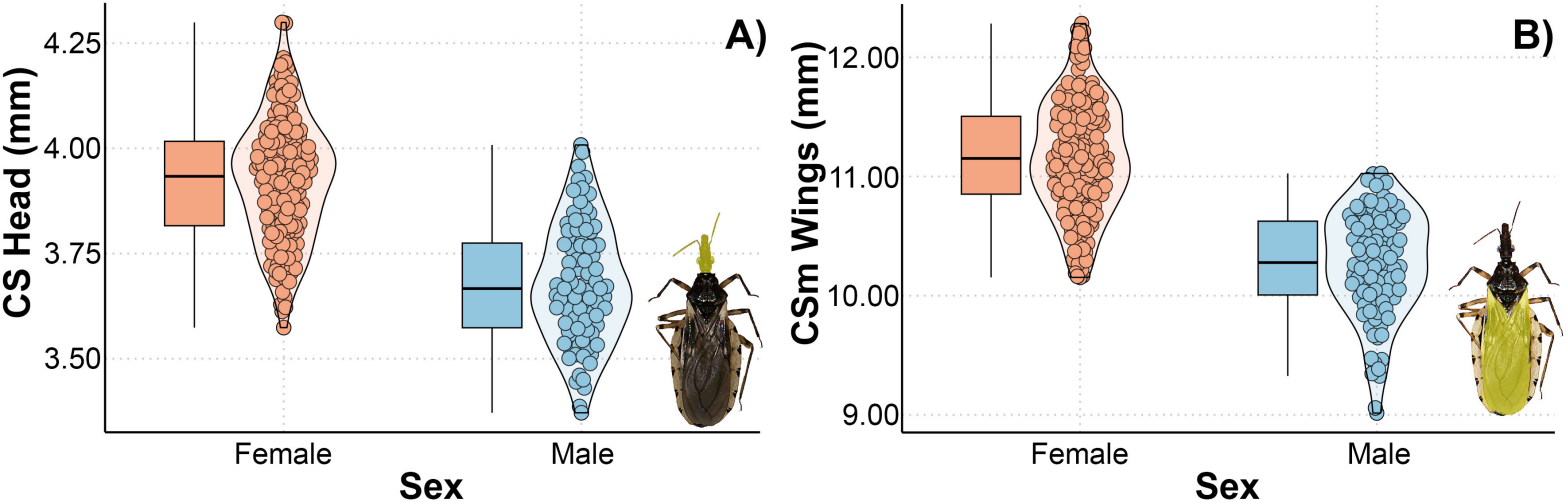
Relationship between landscape metrics at 2400 meters through an anthropization gradient on the centroid size (CS) of the head **(A)** and wings **(B)** of *Triatoma guasayana*. Sex, individual’s sex.

One head and wing centroid size model with ΔAIC < 3 was selected (**Table 3**). In both the head and wings, sex was the only predictor variable that explained size variation. Overall, the females had higher heads and wings than the males (**Figures 6A, B**).

## Discussion

Our study further supports the hypothesis that the fragmentation of the landscape acts as a selective pressure on developmental stability in structures related to flight dispersion (3). Previous research by Fiad et al. (45) demonstrated that anthropization influences *T. garciabesi* and *T. guasayana*, leading to phenotypic changes across an anthropization gradient. However, the relationship between fragmentation and developmental stability regarding flight dispersal remained unclear. Here, we examine the direct impact of fragmentation on developmental stability related to flight dispersion in two Triatominae species. The primary focus of this study is that habitat fragmentation due to anthropization gradients influences the variation in flight-related traits differently between the two triatomine species studied. These differences can likely be attributed to their life history characteristics and their varying sensitivity to changes in their surrounding landscape. The effects of fragmentation caused by anthropization on morphological traits such as head and wing structure may be more pronounced in *T. garciabesi* than in *T. guasayana*. For *T. garciabesi*, the greater degree of asymmetry in the eyes and head and the greater size of the head indicate a stronger response to landscape fragmentation, particularly in environments with numerous habitat patches and higher landscape complexity. These findings may reflect phenotypic specialization in response to fragmented habitats, supporting the hypothesis that landscape fragmentation influences dispersal decisions by increasing costs. These drive phenotypic specializations that enhance the ability to cross the matrix and travel longer distances (3). In contrast, *T. guasayana* exhibited less pronounced morphological changes, possibly indicating lower sensitivity to habitat fragmentation or a more rigid response to landscape modifications. This variation between the species suggests that the degree of landscape alteration can shape species-specific adaptations, with *T. garciabesi* demonstrating greater sensitivity to environmental pressures. Overall, these results underline the importance of considering the interplay between landscape characteristics when studying morphological adaptations in response to habitat anthropization. This study is the first to examine how landscape fragmentation shapes morphological changes in two triatomine species.

## Developmental stability and morphological adaptation to fragmented habitats in *Triatoma garciabesi*

Our findings reveal that head asymmetry differs significantly between intermediate and low anthropization levels, suggesting that specific environmental pressures may influence developmental stability in particular habitats. Previous studies have detected shape variations between these levels (45), and our results indicate that these differences may be driven by FA rather than symmetrical modifications. This aligns with findings in other Triatominae species, where increased FA has been associated with environmental stress during insect development (review in 30).

Several stress factors may contribute to this pattern, including extreme temperatures, changes in vegetation cover, the quality of food sources from hosts, and exposure to environmental chemicals such as insecticides (45, 64–68). Beasley et al. (69) postulated that the relationship between FA and stress is stronger when the source of stress is anthropogenic. Furthermore, it has been proposed that this species is sensitive to vegetation changes and landscape features (40, 45, 68). In this context, environmental stressors such as changes in the availability of suitable refuges and/or reduced food sources for this species due to landscape anthropization could be key factors. Increasing anthropization in the Arid Chaco has led to the reduction of large extensions of mature forest and a decline in avian biodiversity (70–73). This process directly affects *T. garciabesi* by reducing the availability of both shelters and bird hosts, which serve as a crucial blood source for this species. The resulting habitat degradation could further exacerbate the stress experienced by individuals in more fragmented environments. These stress factors, associated with habitat loss and reduced food availability, may directly impact the developmental stability of specific morphological structures. Our results indicate that habitat fragmentation increases FA in *T. garciabesi*, particularly in the anteocular region, where asymmetry was greatest in highly fragmented landscapes. In contrast, compound eyes tended to remain more symmetrical in those environments. FA-driven changes in head morphology involved compression of the anteocular region, resulting in more compact heads with greater ocular convexity. These characteristics resembled those described for macropterous individuals of *T. guasayana*, which tend to have shorter anteocular distances and smaller head sizes than micropterous specimens (23, 24, 74). These changes could suggest potential flight-related adaptations. Alternatively, such asymmetrical modifications may reflect developmental instability rather than true adaptation. Since FA reflects perturbations during development, it may disrupt the integration between eye shape and head structure, potentially affecting visual performance and limiting effective dispersal in fragmented landscapes. This altered head morphology may partially explain the observed reduction in population density from low to intermediate and highly anthropized areas (45).

Beyond the head, we also found significant differences in wing shape symmetry between intermediate and low anthropization levels. This suggests that habitat fragmentation may influence this trait through selective pressures that disrupt the canalization process (22). Additionally, the relationship between wing symmetry and patch number suggests an adaptation process to fragmentation. In highly fragmented landscapes with intermediate levels of anthropization, an adaptive response may arise, favouring individuals with traits that enhance long-distance flight. In contrast, phenotypic characteristics may be better suited for short-range dispersal in less fragmented environments, corresponding to low or high anthropization levels. Despite these differences in wing shape, wing asymmetry remained stable across the fragmentation gradient. This persistence suggests that wing asymmetry may be relatively conserved and potentially useful as an indicator of local environmental adaptation (30). Its maintenance could also be driven by developmental constraints or functional factors such as flight, which may restrict morphological variability and regulate developmental stability (3, 75).

Although the centroid size of the head and wings in *T. garciabesi* was associated with landscape metrics, the weak relationship coefficients suggest these effects are not biologically significant. Only sex emerged as a relevant factor, indicating sexual dimorphism in those traits and suggesting that females were larger than males. The existence of sexual dimorphism is well-known in *Triatominae* (76, 77). According to Cox et al. (78) and Fairbairn et al. (79), sexual dimorphism may be the result of ecological and reproductive pressures. In triatomines, it could be related to feeding habits, population density, habitat, or climatic changes (76, 80, 81). Some authors have proposed that insects with larger heads and wings have greater dispersal capacity (3, 82, 83). In the context of the sexual dimorphism observed in this study, this could enhance flight performance in mated females.

## Landscape fragmentation and morphological resilience in *Triatoma guasayana*

Head shape symmetry in *T. guasayana* differed significantly between low and intermediate levels of anthropization, as well as between low and high levels. Additionally, variations in both the symmetric and FA components of head shape drove differences between low and intermediate anthropization levels. In contrast, differences between low and high anthropization levels were solely attributed to changes in the FA component. The FA component of wing shape also differed significantly between low and intermediate levels of anthropization. Previous studies have reported head and wing shape differences between these levels of anthropization, suggesting that such changes may reflect morphological adaptations to enhance flight dispersal in more fragmented landscapes (45). However, the weak correlation between landscape metrics and both the symmetric and FA shape components suggests that landscape fragmentation might indirectly influence phenotypic plasticity.

Fragmentation may reduce the variation of local conditions within patches while increasing variation among patches (4). These local conditions create patch-specific selective pressures that act on the phenotypic plasticity of the populations inhabiting them, potentially altering dispersal propensities (3). The less pronounced morphological changes observed in *T. guasayana* compared to *T. garciabesi* align with previous findings. Our results, along with those of Fiad et al. (84, 45), suggest that, in this species, seasonality may play a more significant role than anthropization metrics in shaping flight-related traits.

In a study conducted in the arid Chaco of La Rioja province, *T. guasayana* was more frequently found in areas with low vegetation cover near houses (≤100 meters) and intermediate vegetation cover within a 1000 meters radius. Our findings indicate that both the symmetric and asymmetric components of shape begin to associate with landscape metrics beyond 2400 meters from houses. This suggests that these shape components influence dispersal beyond this distance, to fragmentation metrics such as PLAND, number of patches, aggregation index, and landscape shape index. These associations may reflect a direct impact of fragmentation on developmental stability related to flight dispersal. Wing asymmetry mechanically compromises flight performance and function in insects (85–87).

*Triatoma guasayana* exhibits a dispersal strategy predominantly adapted for flight, enabling a greater number of individuals to traverse the landscape and more frequently invade rural dwellings (36). Thus, individuals with lower asymmetry in flight-related traits may have an advantage for dispersal. Individuals of *T. guasayana* that develop during winter and emerge as adults in early summer, within a 2400 meters radius around houses, are the most likely to fly and invade households in the Arid Chaco. In these cases, domestic animals serve as a key food resource. The availability of such resources, combined with the surrounding semi-wild environments, may favour the persistence of wild foci that invade households during warmer seasons. The relatively lower evidence of developmental instability within the 2400 meters radius could enable these insects to explore different environments until suitable ones are identified for establishment and colony growth. This reflects greater resilience to environmental pressures associated with anthropization. This pattern may also explain why, in this area, captures of *T. guasayana* were three times higher than those of *T. garciabesi*, and why their density index remained similar across anthropization levels (45).

In summary, *T. guasayana* appears to exhibit greater morphological stability and adaptability to fragmented environments compared to *T. garciabesi*. Its flight-oriented dispersal strategy and lower developmental instability near human dwellings may enhance its ability to exploit semi-wild and domestic ecotones. This resilience may partially explain its higher prevalence across the anthropization gradient and its potential role as a persistent vector in rural areas of the Arid Chaco. These findings have important epidemiological implications, as they suggest that *T. guasayana* may maintain high dispersal potential and population densities even in fragmented landscapes. This, in turn, could facilitate its role as a bridge vector between sylvatic and domestic environments, increasing the risk of Chagas disease transmission in human-occupied areas.

## Conclusion

Our results show that habitat fragmentation imposes differential selective pressures on *Triatoma garciabesi* and *T. guasayana*, leading to divergent dispersal behaviour. *T. garciabesi* exhibits higher developmental instability and marked morphological shifts in fragmented landscapes, suggesting a more plastic and potentially more energetically costly dispersal syndrome. In contrast, *T. guasayana* maintains more stable morphological traits across the fragmentation gradient, which may reflect a more conservative or less environmentally responsive dispersal strategy. These species-specific responses highlight the role of habitat structure in shaping dispersal-related traits and point out the importance of incorporating landscape configuration into our understanding of the adaptive and movement dynamics of these vector species.

## Data Availability

The raw data supporting the conclusions of this article will be made available by the authors, without undue reservation.

## Supplementary material

Supplementary Figure 1 Effect of landscape metrics, measured at different radius (200 to 3000 m) around of each visited dwellings, on different morphological traits of *Triatoma garciabesi*. Each point in the figure represents the mean Pearson correlation coefficient (r) of linear regressions, Effect Size (Z) and *p*-value associated.

Supplementary Figure 2 Effect of landscape metrics, measured at different radius (200 to 3000 m) around of each visited dwellings, on different morphological traits of *Triatoma guasayana*. Each point in the figure represents the mean Pearson correlation coefficient (r) of linear regressions, Effect Size (Z) and *p*- value associated.
