# Supplementary figures and images for: Breaking symmetry: effects of habitat disturbance on flight-related traits of two Triatominae species
Source: Front Insect Sci. 2025 Sep 8;5:1651021. doi: 10.3389/finsc.2025.1651021 (PMC12452036; doi:10.3389/finsc.2025.1651021)

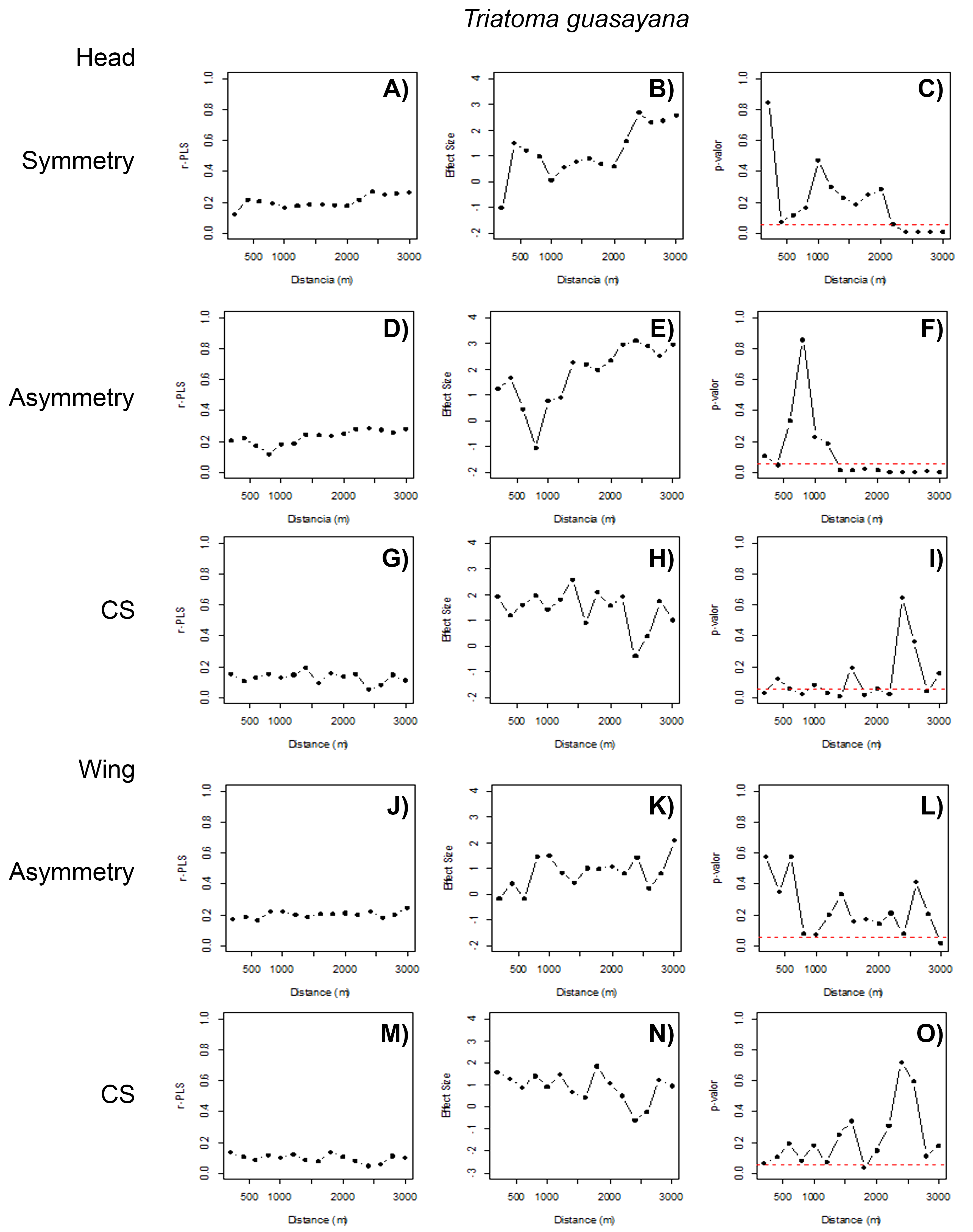

Supplement: Supplementary Figure 1 — Effect of landscape metrics, measured at different radius (200 to 3000 m) around of each visited dwellings, on different morphological traits of Triatoma garciabesi. Each point in the figure represents the mean Pearson correlation coefficient (r) of linear regressions, Effect Size (Z) and p-value associated. [file Image1.tif]

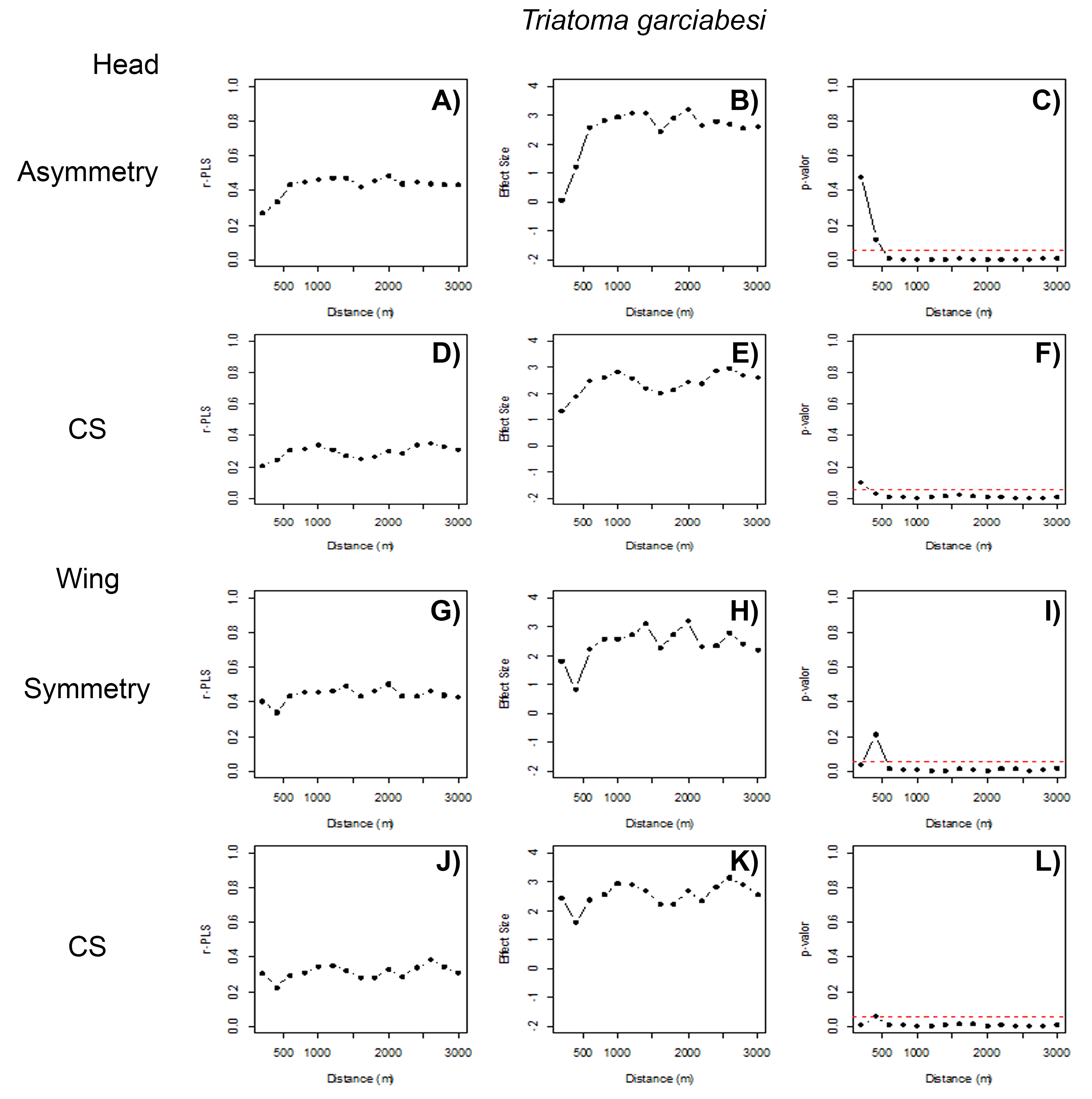

Supplement: Supplementary Figure 2 — Effect of landscape metrics, measured at different radius (200 to 3000 m) around of each visited dwellings, on different morphological traits of Triatoma guasayana. Each point in the figure represents the mean Pearson correlation coefficient (r) of linear regressions, Effect Size (Z) and p- value associated. [file Image2.tif]
